# STAT6: Multidimensional analysis from hematopoietic immune regulation to pathogenesis mechanisms and therapeutic targets in hematologic malignancies

**DOI:** 10.1007/s00277-026-06932-2

**Published:** 2026-03-16

**Authors:** Shuni Zhang, Shuzhen Xiong, Xinyang Liu, Jiajia Cao, Ningning Yue, Chongyang Wu

**Affiliations:** Department of Hematology, Lanzhou University Second Hospital, Lanzhou, China

**Keywords:** STAT6, Hematological malignancies, Immune regulation, Chemotherapy resistance

## Abstract

Signal Transduction and Activation of Transcription 6 (STAT6) is a key molecule in the Janus Kinase/Signal Transducer and Activator of Transcription (JAK/STAT) signaling pathway. Primarily induced by Interleukin-4(IL-4) and Interleukin-13(IL-13), STAT6 undergoes phosphorylation and translocates to the nucleus, where it regulates gene transcription. While the abnormal activation and oncogenic functions of STAT6 in various solid tumors are well documented, its precise biological role in the hematopoietic system has not yet been systematically investigated. Compared with solid tumors, STAT6 activation in hematological malignancies occurs with the Type 2 T helper cell (Th2-type) immune microenvironment that it mediates, which is probably a unique pathogenic mechanism. This review, therefore, delves into the multifunctionality of STAT6 within the hematopoietic system (including viral infection and immune regulation) and hematologic malignancies, as well as its impact on chemotherapy resistance. The aim is to provide a theoretical basis for understanding the biological functions of STAT6 and developing new therapeutic approaches.

## Introduction

Signal Transduction and Activation of Transcription 6 (STAT6) is a 233-amino-acid member of the STAT protein family. When the cytokines such as Interleukin-4 (IL-4) and Interleukin-13 (IL-13) bind to their corresponding receptors, STAT6 becomes activated and phosphorylated. Two STAT6 molecules then form a dimer and translocate into the nucleus. By binding to a specific sequence in the promoter region of target genes, STAT6 regulates the transcription of downstream genes. This mechanism gives STAT6 a central role in various pathological processes, including cancer, inflammation, allergic diseases, and autoimmune disorders^1^. While altered STAT4 expression is prevalent in many tumors and autoimmune diseases, dysregulation of the STAT6 signaling pathway is particularly prominent in classical Hodgkin lymphoma (cHL) and non-Hodgkin lymphoma (NHL)^2^. Research suggests that intranuclear mutations in STAT6 exhibit a familial inheritance pattern. These mutations enhance STAT6’s nuclear localization capacity, DNA-binding affinity, and spontaneous transcriptional activity^2^,^3^. Subsequently, they cause autonomous functional alterations in cells, amplifying signal transduction intensity and producing abnormal immune cell phenotypes, which promote the initiation and progression of lymphoma.

The STAT6 protein functions through the synergistic action of its six core domains (ND, Coiled-Coil domain (CCD), DNA-Binding domain (DBD), Linker domain (LK), Src Homology 2 domain (SH2), Transactivation domain (TAD)) and a key tyrosine residue (Y641). Activation begins at the Y641 site, where JAK kinases are recruited to phosphorylate STAT6. The phosphorylated Y641 site on this STAT6 monomer then recognizes and binds to the SH2 domain of another STAT6 monomer, forming a stable head-to-tail dimer. Finally, this dimer translocates into the nucleus, driven by its TAD domain. Once in the nucleus, the DBD domain of the STAT6 protein recognizes and binds to the specific palindromic sequence [TTC (N)2–4GAA], thereby anchoring the complex to the regulatory region of the target gene. During this process, the CCD domain provides an adhesive scaffold for other transcription factors, enhancing the transcriptional activity and stability of the complex. The LK domain maintains the coordination conformation between the DBD and SH2 domains to ensure their functional connection, while the ND domain mediates the formation of a tetramer from two dimers to enhance the stability and specificity of DNA-binding^4^. 

**Fig. 1:**
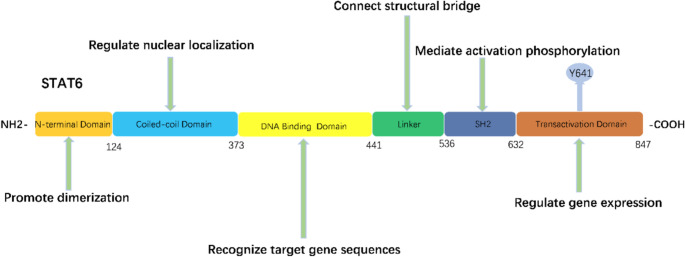
Structural diagram of STAT6, showing amino acid residue numbers and functional components. The STAT6 molecule comprises an N-terminal coiled-coil domain (CCD), a DNA-binding domain (DBD), an SH2 domain, and a C-terminal transactivation domain (TAD). Through the coordinated action of these domains, STAT6 is able to carry out the entire process, from receiving signals and dimerizing to nuclear translocation and finally regulating gene expression.

Figure Legends: Figure 1 was created with Microsoft PowerPoint (version 2021). Previous studies indicate that STAT6 is a key mediator in various classic allergic diseases, such as Atopic dermatitis^5^, Asthma, Allergic rhinitis^3^, Eosinophilic gastrointestinal diseases (EGIDs)^6^ and Lymphoproliferation disorders 7, among others. In addition, the progression of non-allergic complications accompanying the STAT6-related diseases includes skin and respiratory tract infections, osteoporosis, renal fibrosis, pathological fractures, short stature, joint hyperextensibility, and hypotrichosis^8^. While most reports have indicated that D419H is the hotspot mutation of STAT6 9, its two activation mechanisms are different: one depends on the IL-4/IL-13/JAK signaling pathway, while the other does not. Notably, both pathways share a common dependency on phosphorylation at the Y641 site^10^. In summary, STAT6 plays a clearly significant role in allergic diseases and immunodeficiency-related disorders. However, its pathological function and mechanisms in hematological malignancies remain formally undefined.

In multiple hematologic tumor studies, STAT6 has been demonstrated to be closely associated with the risk of developing various lymphomas and their prognosis. However, its specific mechanism of action and clinical significance vary depending on the tumor type. In lymphocyte-predominant Hodgkin lymphoma (LPHL), approximately 48.8% (*n* = 43) of cases exhibited positive expression of phosphorylated STAT6 (p-STAT6)^11^. In follicular lymphoma (FL), the frequency of STAT6 mutations is higher, at 57%, and there is a significant positive correlation with CD23 expression (*P* < 0.001)^12^; additionally, patients with STAT6 gain-of-function (GOF) mutations are at a markedly elevated risk of developing follicular lymphoma^9^. Further studies have revealed that, in primary mediastinal large B-cell lymphoma (PMBL), STAT6 is transported as cargo by the export protein Exportin 1(XPO1). The two proteins colocalize and interact in the nucleus, synergistically enhancing the phosphorylation of STAT6 13 and revealing that the STAT6 protein is uniquely activated in certain types of lymphoma. Notably, STAT6 exhibits different functions in the specific hematologic malignancies. In acute lymphoblastic leukemia (ALL), STAT6 deficiency can lead to a reduction in the number of viable cells due to disturbances in the cell cycle^14^, suggesting that STAT6 may be involved in regulating proliferation in this disease. However, conclusions regarding its clinical significance in primary central nervous system lymphomas (PCNSLs) remain inconsistent. Yang et al. 15 reported STAT6 expression in 61.5% of PCNSL patients treated with methotrexate monotherapy, which was associated with shorter survival. In contrast, Karpathiou et al. 16 found no significant association between STAT6 expression and overall survival or multiple tumor molecular markers (B-cell lymphoma 6(BCL6), B-cell lymphoma 2(BCL2), Myelocytomatosis oncogene(MYC), Programmed cell death ligand 1(PD-L1) and Cluster of Differentiation 8(CD8)), showing only a positive correlation with MYC expression (*p* = 0.02). These conflicting results suggest that the prognostic value of STAT6 in PCNSL requires further validation, and its potential significance as an independent prognostic indicator remains unclear.

## The effects of IL-4/IL-13 signalling on STAT6

**Fig. 2:**
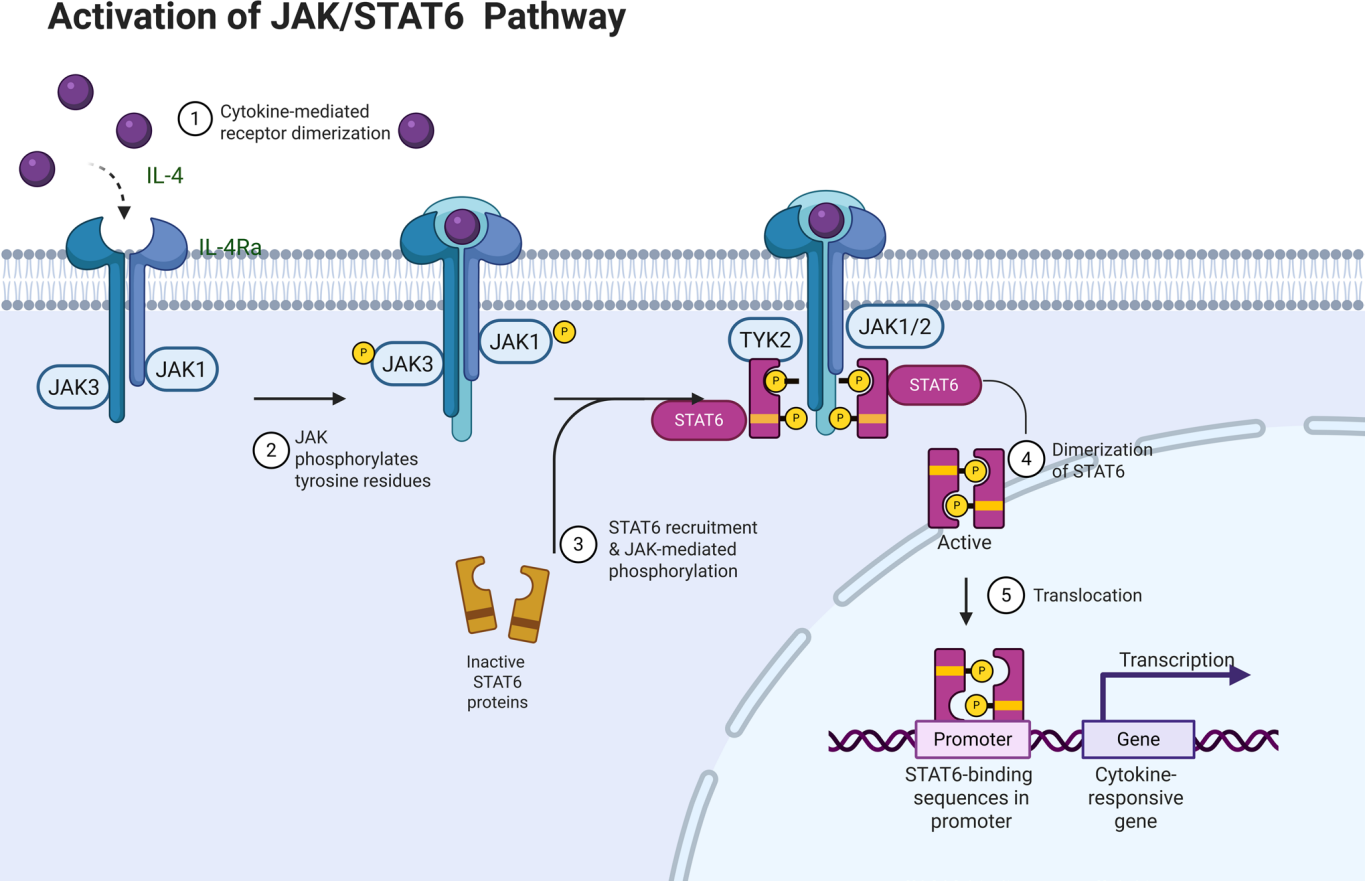
Schematic representation of JAK1/2/3-STAT6 activation. Figure Legends: Figure 2 was created with BioRender.com.

The activation of the STAT6 signaling pathway is primarily regulated by the cytokines IL-4 and IL-13 17. When expressed, these cytokines lead to the intracellular domain of their receptor undergoing autophosphorylation, creating a unique docking site for the SH2 domain of the STAT6 protein, as well as for JAK family kinases. These JAK family members are then recruited and activated to phosphorylate STAT6, leading to STAT6 dimerization and nuclear translocation. There, STAT6 functions as a transcription factor to regulate the expression of downstream target genes, thereby exerting its immunoregulatory role^18^. Notably, researchers observed no significant effect of IL-4 stimulation on total STAT6 protein levels in either wild-type or mutant STAT6 samples^9^. Although the absolute levels of phosphorylated STAT6 (p-STAT6) increased, the ratio of p-STAT6 to total STAT6 remained stable. This indicates that the increase in p-STAT6 levels was not caused by changes in total protein content induced by IL-4 stimulation. Instead, there may be a strict and well-defined intracellular homeostasis buffering mechanism that maintains constant total protein levels during STAT6 activation. (Fig. 2).

## The role of STAT6 in viral infection

Both Epstein-Barr virus (EBV) and human immunodeficiency virus (HIV) have been associated with the development of various hematological disorders. STAT6 is an important innate immune signaling protein that is induced by viral infection. It has different activation modes compared with classic cytokine signaling pathways. Studies using human monocytic leukemia cells (THP-1) reveal that, under unstimulated conditions, STAT6 is diffusely distributed throughout the cytoplasm. However, upon infection with RNA or DNA viruses, its DBD domain interacts with the C-terminal region of the endoplasmic reticulum IFN stimulator (STING) (also known as MITA/ERIS), leading to the co-localization of STAT6 and STING, and their redistribution to the perinuclear region. This ultimately induces the translocation of the STAT6 protein into the nucleus. Furthermore, STAT6 activation during viral infection does not depend on JAK1 or IL-4, but rather requires the involvement of the adaptor proteins STING, TANK-binding kinase 1 (TBK1), and the RNA viral mitochondrial antiviral signaling protein (MAVS)^10^.

Further studies revealed that the chemokine C-C Motif Chemokine Ligand 2 (CCL2) initiates the regulation of STAT6 phosphorylation in infected cells, which occurs before the phosphorylation of other STAT family members. This suggests that STAT6 may serve as a precursor transcription activator precursor in this process. While this response is independent of the classical JAK/STAT pathway, virus-induced phosphorylation at the STAT6 Y641 site is essential for its activation. Furthermore, the study revealed that STING and MAVS do not function in the canonical STAT6 pathway. Taken together, these findings reveal a virus-specific STAT6 activation pathway that is independent of cytokines and is initiated via the STING-MAVS signaling axis. This pathway selectively regulates a specific set of genes involved in immune cell homing. This modulates chemokine expression and immune cell infiltration patterns, ultimately reshaping the antiviral immune microenvironment.

## The role of STAT6 in immune cells

Gain-of-function mutations in STAT6 induce multiple immunological effects, including significant increases in B cells, eosinophils, and mast cells. Concurrently, sustained STAT6 activation promotes Th2 differentiation in helper T cells and positively regulates immunoglobulin class switching to immunoglobulin E (IgE). These phenomena demonstrate the critical role of STAT6 in maintaining normal immune function. Furthermore, studies confirm that STAT6 can enhance the gene expression of chemokines CCL2, CCL20, and CCL26 independently of JAK1 gene upregulation, thereby mediating the targeted recruitment of immune cells to infection sites^10^. This finding enhances our understanding of the mechanisms underlying STAT6’s non-canonical signaling pathways in immune regulation.

### Effects of STAT6 on B cells

The IL-4/STAT6 signaling pathway plays a central regulatory role in B cell proliferation, differentiation, and humoral immune responses. Research indicates that this pathway drives B cell expansion and maturation, and determines their fate as either activated B cells or plasma blasts (PB) by regulating the differential response of precursor B cells to IL-4. Following early IL-4 stimulation, STAT6 signaling typically downregulates or even disappears, prompting B cells to initiate the plasma blastic differentiation programme. However, when the levels of IL-4 remain persistently high, sustained STAT6 activation paradoxically inhibits differentiation and leads to B cells remaining in an activated state^19^, indicating that IL-4 has a counter-regulatory effect on B cell differentiation. Concurrently, gain-of-function mutations in STAT6 further amplify IL-4-driven lymphocyte proliferation, leading to a significant increase in B cell numbers and promoting IgE class switching. This ultimately results in elevated systemic IgE levels^20^,^21^.

At the molecular level, BCL6 induces somatic hypermutation and B-cell receptor (BCR)-mediated degradation of BCL6. However, the IL-4/STAT6 signaling pathway can directly induce the BCL6 transcription, hindering its degradation and enhancing its stability. This further increases the magnitude and duration of the GC response^22^ by enhancing the function of the B cell immune response in vivo. Therefore, this study demonstrates that STAT6 plays a crucial role in integrating B-cell fate determination and functional regulation.

### The effects of STAT6 on T cells

STAT6 plays a critical regulatory role in CD4 + T helper cell differentiation, particularly in determining the fate of Th2 cells. CD4 + T cells can differentiate into distinct subsets, including Th1, Th2, Th17, and Th22 cells. Studies indicate that SARS-CoV-2 infection activates STAT6 signaling pathways in both the cytoplasm and nucleus of lung cells, thereby promoting Th2 cell polarization and contributing to cytokine storm formation. This ultimately leads to an imbalance in Th1/Th2 immune responses^3^. Interestingly, there were no significant differences in the Th17 and Th22 cell subsets of patients with STAT6 gene mutations compared to healthy individuals, which further highlights the specific role of STAT6 in Th2 differentiation.

In terms of the regulatory mechanisms of STAT6 activation, TNF receptor-associated factor 3(TRAF3) enhances IL-4-mediated STAT6 activation in CD4-T cells^23^. Conversely, the protein tyrosine phosphatase Protein tyrosine phosphatase non-receptor type 1 (PTP1B) acts as a negative regulator by directly binding to the IL-4 receptor complex and interfering with JAK1-mediated STAT6 phosphorylation. This suppresses the upregulation of the GATA binding protein 3(GATA3) transcription factor and Th2 cell polarization^23^. Taken together, these findings establish STAT6 as a key mediator of Th2 cell differentiation.

The STAT6 signaling pathway also plays a significant role in T cell responses during the clinical treatment of hematologic malignancies. Bone marrow transplantation (BMT) is the most commonly used treatment for hematological malignancies and can induce a graft-versus-tumor (GVT) effect mediated by the donor T cells in the transplant. However, BMT can also induce graft-versus-host disease (GVHD). Our previous reports have demonstrated that IL-4 can induce the secretion of Th2 cytokines (including IL-4, IL-10, and TGF-β) via activation of the STAT6 signaling pathway, forming a positive feedback regulatory loop. The established Th2-polarized state can enhance the GVT effect, and simultaneously diminish the GVHD response by suppressing the production of pro-inflammatory factors and vascular endothelial growth factor (VEGF) 24. This provides new approaches to preventing tumor recurrence post-transplantation and reducing GVHD-related damage.

### The role of STAT6 in macrophages

Macrophages are a crucial component of the innate immune system and exhibit high functional heterogeneity and plasticity^25^. In the tumor microenvironment, human peripheral blood mononuclear cells can differentiate into macrophages and polarize into either the pro-inflammatory M1 phenotype or the anti-inflammatory M2 phenotype. Studies indicate that STAT6 plays a central regulatory role in M2 polarization of tumor-associated macrophages (TAMs)^26^. Protein blot analysis reveals that STAT6 phosphorylation significantly increases the expression of M2 macrophage gene markers^27^. The process is primarily driven by the IL-4/STAT6 signaling pathway, which encourages macrophages to differentiate into the M2 phenotype, characterized by anti-inflammatory and tissue-repairing properties.

In recent years, significant progress has been made in developing inhibitors that target the regulation of STAT6 and macrophage polarization. The phosphopeptide mimic 37 (PM37) targets explicitly the SH2 domain of STAT6, effectively blocking and reversing the expression of M2 polarization markers^28^. In tumor cell-M2 macrophage co-culture systems, PM37 sensitizes tumor cells to radiation therapy, providing a new strategy to overcome tumor radioresistance. Peroxisome Proliferator-Activated Receptor γ (PPARγ) is a kind of nuclear hormone receptor found in cells that can activate oxidized fatty acids. The IL-4/STAT6 signaling pathway enhances the DNA-binding activity of PPARγ and regulates the lipid metabolism and inflammatory response of PPARγ target cells (macrophages and dendritic cells(DCs)) by expanding the range and intensity of regulated genes^29^.

Further preclinical studies reveal that the Bruton’s tyrosine kinase inhibitor Zanubrutinib can reprogram macrophages from the M1 phenotype to the M2 phenotype by modulating the STAT6 signaling pathway, thereby maintaining immune homeostasis^30^. Concurrently, exosome-based targeted therapeutic strategies demonstrate promising applications. As they are released by cells, exosomes serve as ideal drug delivery vehicles. Research utilizing engineered exosomes to deliver STAT6 antisense oligonucleotides (ASOs) has successfully induced TAMs to reprogram from the M2 phenotype to the pro-inflammatory M1 phenotype, thereby reshaping the tumor microenvironment^31^. These studies provide novel insights and approaches for developing STAT6-targeted tumor immunotherapy.

## The role of STAT6 in hematologic malignancies

Hematologic malignancies are malignant proliferative diseases that originate from the hematopoietic system. They primarily encompass leukemia and lymphoma. Lymphoma can be further classified as Hodgkin lymphoma (HL) or non-Hodgkin lymphoma (NHL). NHL exhibits significant biological and clinical heterogeneity, primarily arising from the malignant transformation of mature B lymphocytes or T lymphocytes. The major subtypes of B-cell lymphomas, based on cellular origin, include diffuse large B-cell lymphoma (DLBCL), follicular lymphoma (FL), and primary mediastinal B-cell lymphoma (PMBL), which have unique clinicopathological features. T-cell-derived subtypes primarily include peripheral T-cell lymphoma (PTCL), cutaneous T-cell lymphoma (CTCL), and the distinct subtype of breast implant-associated anaplastic large cell lymphoma.

### Mechanism of STAT6 in acute lymphoblastic leukemia cells

Acute lymphoblastic leukemia (ALL) is a highly aggressive malignancy that originates in lymphoid progenitor cells and predominantly affects children and the elderly^32^. In the specific genetic subtype of Philadelphia chromosome-positive ALL (Ph + ALL), the characteristic Breakpoint cluster region-Abelson murine leukemia viral oncogene homolog fusion gene/protein (BCR-ABL) fusion protein exhibits two major subtypes: P210 and P190. Research indicates that abnormal STAT6 activation is particularly pronounced in the P190 subtype. This involves Jak2-phosphorylated STAT6 binding to the c-MYC gene promoter as a transcription factor, thereby driving disease progression in Ph+ ALL 33. Comparative cytokine levels in patient cells revealed no significant differences between P190 and P210 cells. This suggests that classical cytokine pathways do not mediate elevated Phosphorylated STAT6 (p-STAT6) in P190 cells, but rather depend on Jak2 pathway activation^34^.

Furthermore, studies confirm that the BCR-ABL kinase regulates STAT6 activation. When p-STAT6 is inhibited, ALL cells undergo cycle rearrangement, which manifests as G2/M phase arrest and a significant decrease in the proportion of S phase cells, accompanied by an increase in apoptosis rates. Analysis of clinical samples also revealed that patients with significantly reduced STAT6 levels at diagnosis exhibited higher rates of leukemic cell apoptosis upon relapse^35^. Collectively, these findings suggest that STAT6 is a key molecule in the mediation of malignant proliferation and poor prognosis in Ph + ALL. Furthermore, they indicate that targeted inhibition of p-STAT6 could be an effective strategy for suppressing P190 cell proliferation.

In B-cell precursor acute lymphoblastic leukemia (BCP-ALL), the expression of the IL-13/STAT6 signaling pathway greatly upregulates cytidine deaminase Activation-induced cytidine deaminase (AID, encoded by the AICDA gene) in germinal center B cells and helper T cells^36^, which are associated with higher clonal mutation burdens and poorer clinical outcomes, This suggests an extensive pathogenic role for STAT6 in ALL subtypes.

### Mechanism of STAT6 in hodgkin lymphoma

HL is a lymphoid malignancy with unique pathological features, accounting for 15–25% of all lymphomas^37^. The expression status of STAT6 is used as a reference in the empirical classification of cHL. STAT6 positivity typically indicates the mixed cellularity subtype or lymphocyte-rich subtype, whereas STAT6 negativity is more commonly observed in nodular lymphocyte-predominant HL 38. In cHL, Hodgkin/Reed–Sternberg (HRS) cells express high levels of JAK2^11^, components of the IL-13 signaling pathway, phosphatase of regenerating liver-3 (PRL-3) 39, and lymphotoxin α (LTA)^40^, among other factors. Approximately 85% of HRS cells can harbor this cytokine network, which can phosphorylate STAT6 and then promote tumor cell survival and migration^41^. Consequently, p-STAT6 detection is valuable for distinguishing cHL from certain non-Hodgkin lymphomas.

Notably, HRS cells in cHL secrete thymic and activation-regulated chemokine (TARC), also known as C-C motif chemokine ligand 17 (CCL17). This factor attracts Th2 cells to the tumor microenvironment. These cells then secrete cytokines such as IL-4, which activate the STAT6 signaling pathway and promote further TARC secretion. This positive feedback loop continuously attracts eosinophils and establishes an immunosuppressive microenvironment, ultimately facilitating immune evasion in HL 42. In exploring therapy options, it was found that the histone deacetylase (HDAC) inhibitor Vorinostat not only downregulates STAT6 mRNA expression and inhibits tumor proliferation, but also directly induces cell cycle arrest and apoptosis in HL cells^43^.

### The mechanism of STAT6 in diffuse large B-cell lymphoma

DLBCL is the most common subtype of NHL, exhibiting high genetic heterogeneity and mutational diversity. The recurrence rate is as high as 40%, with an inferior prognosis and high mortality following relapse^2^. Among the gene features associated with DLBCL recurrence, the STAT6 D419N mutation is a critical gain-of-function site. This mutation induces the high expression of multiple genes related to cell proliferation and chemotaxis^18^. This reveals a potential mechanism for sustained signaling pathway activation during DLBCL recurrence: STAT6 activation is driven not by exogenous cytokines such as IL-4, but by the mutation itself within the STAT6 gene in the cell nucleus^44^.

Further studies revealed that the tumor microenvironment of STAT6-positive patients contained significantly higher proportions of CD4 + T cells than that of STAT6-negative patients. This suggests that STAT6 may recruit helper T cells to the tumor site via chemotaxis in order to modulate antitumor immune responses. As STAT6 mutations are frequently observed in germinal center B-cell-like (GCB) subtypes^41^, researchers constructed a GCB subtype lymphoma cell line harboring the STAT6 D419N mutation via plasmid construction. Under IL-4 co-culture conditions that simulate STAT6 overexpression, it was found that the STAT6 D419N mutation itself lacks constitutive activity^18^ and its growth-promoting effects depend on IL-4 stimulation^45^. Notably, although exogenous IL-4 does not significantly affect the phosphorylation level of STAT6 D419N, the full transactivation function of this mutant protein still requires IL-4 participation^46^. These seemingly contradictory experimental results collectively indicate that, in DLBCL, the STAT6 D419N mutation is the core intrinsic factor affecting its function, while external cytokine stimulation plays a synergistic regulatory role. In terms of therapy, the epigenetic modulator azacitidine could suppress JAK2-STAT6 signaling activity by inhibiting the hypermethylation of miR-518a-5p. This inhibitor suppressed DLBCL cell proliferation and invasion, promoted apoptosis, and induced cell cycle arrest^47^, making it an ideal targeted therapeutic agent.

### The mechanism of STAT6 in follicular lymphoma

FL, the second most common NHL, is closely associated with the IL-4/IL-4R-activated JAK–STAT6 signaling pathway in its development^48^. This pathway mediates tumorigenesis in stage I FL patients by modulating BCL2 protein expression through BCL2 translocation^49^, which ultimately promotes CD23 overexpression. Consequently, CD23 expression serves as an alternative biomarker for STAT6 pathway activation^50^. In this process, Poly(ADP-ribose) polymerase family member 14(PARP14) acts as a co-activator that binds to STAT6, thereby enhancing the transcriptional activity of this pathway and promoting the regulation of STAT6 by downstream target genes^46^.

Patients with STAT6 mutations exhibit distinct molecular characteristics compared to those with wild-type STAT6: their STAT6 activation does not depend on tyrosine phosphorylation at the Y641 site^9^, yet they still show high expression of IL-4-responsive genes^51^, accompanied by shorter progression-free survival^52^, reflecting the functional heterogeneity of STAT6 mutations in FL pathogenesis. Further studies have revealed that both IL-4 and BCR signaling activate the mTOR pathway. This process is inhibited by CREB-binding protein (CREBBP) but enhanced by STAT6 53, indicating that STAT6 promotes tumor cell growth via mTOR signaling.

In addition to acting directly on tumor cells, STAT6 plays an active role in remodeling the FL tumor microenvironment (TME). For instance, it recruits CD4 + T cells to infiltrate tumor regions by upregulating the CCL17 chemokine, modulates redox homeostasis and protein disulfide bond formation via Quiescin sulfhydryl oxidase 1 (QSOX1), and influences apoptosis through Nuclear factor, interleukin 3 regulated (NFIL3). In summary, STAT6 mutations play a central role in FL pathogenesis and promote the disease process by multidimensionally regulating the tumor microenvironment.

### Mechanism of STAT6 in primary mediastinal B-cell lymphoma

PMBL is a distinct subtype of DLBCL characterized by unique clinical and pathological features. Its molecular hallmarks primarily involve the abnormal expression of STAT6 and BCL6 54. In PMBL patients, the incidence of STAT6 mutations is relatively low, ranging from approximately 33% to 36%^44^. The disease is characterized by an earlier age of onset and a male predominance.

At the molecular level, PMBL patients commonly exhibit high JAK2 expression 11, with 91% of cases accompanied by Suppressor of cytokine signaling 1(SOCS1) gene mutations^44^. These SOCS1 mutations primarily disrupt normal negative feedback regulation through somatic hypermutation, leading to sustained phosphorylation and activation of STAT6. Interestingly, unlike in FL, CD23 expression in PMBL patients shows no significant correlation with STAT6 mutation status (*p* = 0.13) 55.

STAT6 gene mutations primarily occur in the exons encoding the DNA-binding domain. Approximately 35% of PMBL patients exhibit substitution mutations, predominantly at the D419 site^56^,^57^. These mutations predominantly occur at T: A base pairs within the DBD and SH2 domains^58^, primarily affecting aspartic acid (Asp) residues, followed by glutamic acid (Glu) residues^8^. Analysis of the large-scale population genome database (gnomAD) revealed that the mutation rate of STAT6 in the general population is close to zero and that all identified STAT6 mutations have high Combined Annotation-Dependent Depletion (CADD) scores (> 20 points). Collectively, this evidence supports the idea that both germline and somatic gain-of-function variants in STAT6 independently contribute to the pathogenesis of PMBL.

However, recent studies have demonstrated that STAT6 activation can also be induced by mutations. This finding highlights the distinct nature of PMBL in the regulation of STAT6 signaling compared to other lymphoma subtypes. More importantly, recent studies have reported that the region encoding the STAT6 DNA-binding domain is a major hotspot for recurrent mutations in PMBL^59^, with this molecule playing a central role in PMBL pathogenesis.

### The mechanism of STAT6 in peripheral T-cell lymphoma and T-Lymphoblastic Lymphoma (T-LBL)

PTCL constitutes a group of T-lymphocyte malignancies characterized by significant heterogeneity^60^. The presence of Hodgkin/Reed-Sternberg (HRS)-like cells in the tumor microenvironment often complicates differential diagnosis with other cells that are morphologically similar to HRS cells in cHL. To solve this problem, the study analyzed the molecular characteristics of HRS cells in PTCL and cHL. The study found that HRS-like cells in PTCL exhibited significantly lower expression levels of STAT6 (*P* < 0.001) and p-STAT6 (*P* < 0.001), a difference which was statistically significant^61^. This indicates that the expression status of STAT6 holds important clinical value in differentiating between the two conditions.

.

Furthermore, studies demonstrate that high activation of the JAK1/2-STAT6 signaling pathway correlates positively with favorable progression-free survival (PFS) in patients with T-lymphoblastic lymphoma (T-LBL), the second most common childhood NHL. Therefore, the activity level of the JAK1/2-STAT6 pathway can be used to predict the prognosis of patients with T-LBL, providing a basis for the precise treatment of this disease.

### The mechanism of STAT6 in cutaneous T-Cell Lymphomas (CTCL)

The most common clinical subtypes of CTCL, Sezary syndrome (SS) and mycosis fungoides (MF), exhibit a close association between their pathogenesis and abnormal activation of the STAT6 signaling pathway. Research indicates that SS/MF tumor cells can produce high levels of cytokines, such as IL-13 and IL-19, via autocrine mechanisms, thereby inducing sustained activation of STAT6. Pharmacological inhibition of STAT6 expression greatly downregulates related signaling pathways, including those involved in cell cycle progression, DNA repair, and Th2 cytokine production. This results in tumor cell cycle arrest at the G1/G2 phase and effectively suppresses the proliferative activity of malignant lymphocytes^62^.

Additionally, STAT6 stimulates Th2 cells to generate IL-4 and IL-13, These cytokines then direct the polarization of TAMs towards the M2 phenotype. This alters their unique gene expression patterns, fosters the development of an immunosuppressive microenvironment, and increases tumor invasiveness. Notably, STAT6 also recruits additional immunosuppressive cells to the tumor microenvironment by regulating the expression of chemokines such as CCL17, CCL13, and CCL8 63. At the same time, STAT6 induces the expression of systemic and local immunosuppressive factors, such as TGF-β, thereby enhancing the immunosuppressive functions of TAMs^64^. These mechanisms collectively induce an immunosuppressive state in CTCL and provide an experimental basis for inhibiting the STAT signaling pathway in advanced cutaneous T-cell lymphoma.

### Mechanism of STAT6 in breast implant-associated anaplastic large cell lymphoma

**Fig. 3:**
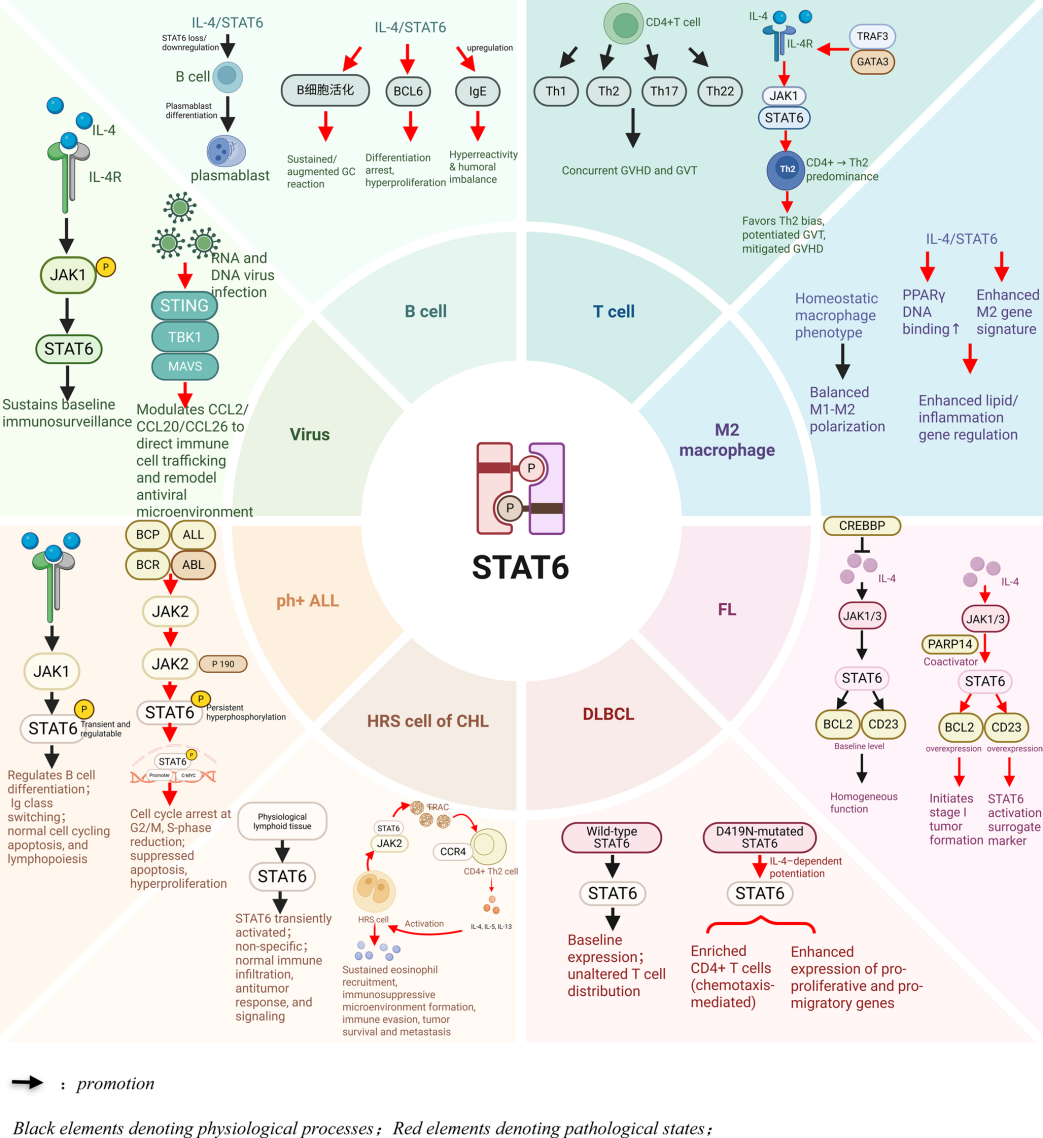
Mechanisms of STAT6 in Different Cell Types, Viral Infections, and Related Diseases.

Breast implant–associated anaplastic large cell lymphoma (BIA-ALCL) is a CD30-positive, ALK-negative peripheral T-cell lymphoma. Immunohistochemical studies reveal that approximately 90% of BIA-ALCL cases express IL-13 65. This cytokine can mediate the downstream STAT6 signaling pathway and promote the survival and proliferation of tumor cells. Subsequent experiments also found that CD30-targeted therapy can significantly reduce the expression of IL-13 in BIA-ALCL and inhibit the growth of tumor cells. Additionally, experimental studies suggest that the STAT6-specific inhibitor AS1517499 can reduce the survival rate of BIA-ALCL cell lines by up to 80% 66. These results indicate that the STAT6 signaling pathway plays a significant role in the pathogenesis process of BIA-ALCL and provides a basis for the use of STAT6 inhibitors in the treatment of this disease. (Fig. 3).

Figure Legends: Figure 3 was created with BioRender.com. Schematic illustration of signal transducer and activator of transcription 6 (STAT6) signaling pathways across multiple cell types and pathological conditions. **Center**: Domain structure of STAT6 protein featuring phosphorylation sites (P). **B cell compartment (upper left)**: Canonical IL-4/STAT6 signaling through JAK1 sustains baseline immunosurveillance, regulating B cell differentiation, immunoglobulin class switching, and normal lymphopoiesis. Sustained IL-4/STAT6 activation leads to B cell activation, BCL6 upregulation, and IgE hyperreactivity, resulting in differentiation arrest and humoral imbalance. **Viral infection (left)** : Viral infection activates STAT6 via the non-canonical STING-TBK1-MAVS axis, independent of JAK1, modulating chemokine expression (CCL2/CCL20/CCL26) to direct immune cell trafficking. **T cell compartment (upper right)**: IL-4/STAT6 signaling promotes CD4 + T cell differentiation toward Th2 predominance, facilitated by TRAF3 and GATA3. This Th2 bias favors graft-versus-tumor (GVT) effects while mitigating graft-versus-host disease (GVHD) in bone marrow transplantation settings. **M2 macrophage compartment (right)**: IL-4/STAT6 signaling drives M2 polarization through PPARγ DNA binding enhancement, resulting in altered lipid metabolism and inflammatory gene regulation compared to homeostatic M1-M2 balanced phenotypes. **Follicular lymphoma (FL) (lower right)**: Wild-type STAT6 maintains baseline BCL2 and CD23 expression. PARP14 serves as a STAT6 coactivator, promoting BCL2 and CD23 overexpression, which initiates stage I tumor formation and serves as a STAT6 activation surrogate marker. CREBBP negatively regulates this pathway. **Diffuse large B-cell lymphoma (DLBCL) (bottom**): Wild-type STAT6 shows baseline expression with unaltered T cell distribution, whereas D419N-mutated STAT6 exhibits IL-4-dependent potentiation, enriched CD4 + T cell infiltration via chemotaxis, and enhanced expression of pro-proliferative and pro-migratory genes. **Classical Hodgkin lymphoma (cHL) (lower left)**: In physiological lymphoid tissue, STAT6 undergoes transient activation with normal immune function. In Hodgkin/Reed-Sternberg (HRS) cells, constitutive JAK2 activation drives persistent STAT6 phosphorylation, establishing a positive feedback loop involving TARC secretion and CCR4 + Th2 cell recruitment. This results in sustained eosinophil recruitment, immunosuppressive microenvironment formation, immune evasion, and tumor survival. **Philadelphia chromosome-positive acute lymphoblastic leukemia (Ph + ALL) (left)**: Normal BCP-ALL shows transient, regulatable STAT6 phosphorylation. The P190 BCR-ABL subtype exhibits JAK2-mediated persistent STAT6 hyperphosphorylation, promoting c-MYC transcription, cell cycle arrest at G2/M phase, S-phase reduction, suppressed apoptosis, and hyperproliferation.

## Role of STAT6 in chemotherapy resistance in hematologic malignancies

Targeting STAT6 to reverse treatment resistance is an important direction for precision therapy in hematologic malignancies, yet its potential value and intervention strategies show significant heterogeneity or even contradictory effects depending on the disease type.

In DLBCL, lymphoma cells carrying the STAT6 D419N mutation do not exhibit altered responsiveness to any single agent in the standard R-CHOP regimen (rituximab, cyclophosphamide, doxorubicin, vincristine, and prednisone) 18, suggesting that the role of STAT6 in regulating chemotherapy resistance in DLBCL may be limited. In contrast, activation of STAT6 clearly promotes the resistant phenotype in other diseases. For example, in AML, overexpression of heme oxygenase-1 (HO-1) can induce chemotherapy resistance in leukemic cells harboring Fms-like tyrosine kinase 3 (FLT3) internal tandem duplication (ITD) mutations via the IL-4/STAT6 pathway, and this effect can be reversed by the novel FLT3 tyrosine kinase inhibitor Gilteritinib^67^. In the tumor microenvironment of CLL, a large amount of IL-4 derived from T cells activates the JAK1/3–STAT6 axis and the downstream atypical Nuclear Factor kappa-B (NF-κB) pathway, upregulating anti-apoptotic proteins and thereby enhancing tumor cell survival and resistance^68^.

Conversely, in some leukemia models, inhibiting STAT6 signaling can effectively enhance the efficacy of chemotherapy. Ruxolitinib can inhibit the JAK1/2–STAT6 pathway and, when combined with dexamethasone, synergistically reverses the resistance of T-cell acute lymphoblastic leukemia (T-ALL) cell lines to glucocorticoids^69^. In B-cell acute lymphoblastic leukemia (B-ALL), STAT6 plays a dual role in promoting oncogenesis and regulating chemotherapy sensitivity. Combined inhibition of Insulin-like Growth Factor 1 Receptor (IGF1R) and MAPK/ERK Kinase (MEK) downregulates STAT6 expression, disrupting the STAT6–ERK–NF-κB survival network to enhance sensitivity to cytotoxic drugs^70^,^71^. Downregulation or knockdown of STAT6 can respectively increase the sensitivity of relapsed ALL to cytarabine and the efficacy of chemotherapy in resistant pediatric ALL^35^,^72^. This suggests that targeting STAT6 may become a new strategy to overcome chemotherapy resistance in ALL.

Targeting STAT6 also shows potential in lymphomas. In FL, targeting JAK kinase with Fedratinib^73^, downregulating STAT6 mRNA expression with Vorinostat^74^, or targeting PARP14 to disrupt the STAT6 transcription complex^75^, all successfully induce tumor cell apoptosis. Among these, Vorinostat not only reduces the expression of downstream target genes of STAT6, such as B-cell lymphoma - x Large isoform (Bcl-xL), but also synergizes with chemotherapy agents like doxorubicin and gemcitabine to produce antitumor effects.

It is particularly noteworthy that PMBL and cHL have high biological homogeneity, with the JAK/STAT pathway (especially STAT6) being continuously activated due to recurrent mutations. Recent studies have shown that heat shock protein Heat Shock Protein 110 (HSP110) acts as a molecular chaperone for STAT6, promoting its phosphorylation and stabilization, while Exportin 1 (XPO1) regulates the nuclear-cytoplasmic shuttling of STAT6^76^. Inhibitors targeting both (selinexor or novel HSP110 inhibitors) can suppress STAT6 signaling and exert antitumor effects, and their combined use may produce synergistic effects, offering new therapeutic strategies for these types of lymphomas. (Table 1).

## Limitations and future perspectives

Although the role of STAT6 in drug resistance mechanisms of various leukemias is becoming increasingly clear, its clinical application as a therapeutic target and prognostic marker still faces significant challenges. A phase II clinical trial evaluating the monotherapy of JAK inhibitor ruxolitinib in CLL was prematurely terminated due to severe infections and frequent anemia in patients^77^, highlighting the potential clinical risks of broadly inhibiting the JAK/STAT pathway. Moreover, in highly prevalent tumors such as DLBCL, the prognostic value of STAT6 mutations or expression remains limited, failing to provide stable stratification information. More importantly, the prognostic significance of STAT6 in certain hematologic malignancies, such as PCNSL and ALL, remains controversial, and its clear clinical value needs to be further validated by larger prospective studies.

**Table 1:** Mechanisms of STAT6-mediated drug resistance and therapeutic targeting in hematologic malignancies

Disease Type	STAT6 Role	Mechanism of Action / Key Finding	Targeted Drug/Therapeutic Strategy	Ref
DLBCL	Limited Role	The STAT6 D419N mutation did not alter responsiveness to any single agent in the R-CHOP regimen.	Suggests direct targeting of STAT6 may have limited significance in reversing DLBCL chemotherapy resistance.	^[18]^
AML	Promotes Resistance	HO-1 overexpression mediates resistance in FLT3-ITD cells via the IL-4/STAT6 pathway.	Gilteritinib (FLT3 inhibitor) can reverse this resistance.	^[69]^
CLL	Promotes Resistance	T cell-derived IL-4 in the tumor microenvironment activates the JAK1/3–STAT6 axis and the atypical NF-κB pathway, upregulating anti-apoptotic proteins.	Ruxolitinib (JAK inhibitor) – a Phase II trial was terminated early due to toxicity.	^[70]^
T-ALL	Promotes Resistance	STAT6 activation leads to glucocorticoid resistance.	Ruxolitinib + Dexamethasone combination synergistically reverses resistance.	^[71]^
B-ALL	Dual Role	1. Promotes Survival: Core of a predictive STAT6–ERK–NF-κB survival network. 2. Regulates Sensitivity: Its expression level directly affects sensitivity to drugs like Ara-C.	1. IGF1/R + MEK inhibitor combination sensitizes by co-downregulating STAT6. 2. Direct reduction of STAT6 expression sensitizes to chemotherapy.	^[72, 73]^
FL	Potential Target	STAT6 pathway activation drives tumorigenesis.	1. Fedratinib (targets JAK) 2. Vorinostat (downregulates STAT6 mRNA, synergistic with chemo) 3. Targeting PARP14 (disrupts STAT6-associated complexes)	^[75–77]^
PMBL / cHL	Core Driver Target	The JAK/STAT6 pathway is constitutively activated via recurrent mutations and is finely regulated by HSP110 and XPO1.	1. Selinexor (XPO1 inhibitor) 2. HSP110 inhibitor 3. Combination of both shows synergistic effects.	^[78]^

## **Conclusion**

STAT6, as a key regulatory factor in the immune system, governs the differentiation fate of B cells, T cells, and macrophages. It exerts a vital influence on the genesis and evolution of hematologic malignancies, like leukemia and lymphoma, by modulating cell cycle events, fueling cell proliferation, and remodeling the tumor microenvironment. Current evidence indicates that STAT6 primarily exhibits gain-of-function characteristics in hematologic malignancies. STAT6 plays a key role in immune regulation, tumor formation, and chemotherapy resistance. Targeting it is a promising treatment option for blood diseases and may lead to the development of new targeted drugs.

## Data Availability

Data availability does not apply to this article as no new data were created or analyzed in this study.
